# Web-Based Intervention in Mindfulness Meditation for Reducing Residual Depressive Symptoms and Relapse Prophylaxis: A Qualitative Study

**DOI:** 10.2196/jmir.3129

**Published:** 2014-03-24

**Authors:** Jennifer M Boggs, Arne Beck, Jennifer N Felder, Sona Dimidjian, Christina A Metcalf, Zindel V Segal

**Affiliations:** ^1^Kaiser Permanente ColoradoInstitute for Health ResearchDenver, COUnited States; ^2^University of Colorado BoulderDepartment of Psychology and NeuroscienceBoulder, COUnited States; ^3^University of Toronto ScarboroughDepartment of PsychologyToronto, ONCanada

**Keywords:** mindfulness-based cognitive therapy, online depression, Web-based depression, Internet-based depression, depression relapse prevention, residual depression symptoms, online psychological treatment, qualitative methods

## Abstract

**Background:**

Mindful Mood Balance (MMB) is a Web-based intervention designed to treat residual depressive symptoms and prevent relapse. MMB was designed to deliver the core concepts of mindfulness-based cognitive therapy (MBCT), a group treatment, which, despite its strong evidence base, faces a number of dissemination challenges.

**Objective:**

The present study is a qualitative investigation of participants’ experiences with MMB.

**Methods:**

Qualitative content analysis was conducted via 38 exit interviews with MMB participants. Study inclusion required a current PHQ-9 (Patient Health Questionnaire) score ≤12 and lifetime history ≥1 major depressive episode. Feedback was obtained on specific website components, program content, and administration as well as skills learned.

**Results:**

Codes were assigned to interview responses and organized into four main themes: MBCT Web content, MBCT Web-based group process, home practice, and evidence of concept comprehension. Within these four areas, participants highlighted the advantages and obstacles of translating and delivering MBCT in a Web-based format. Adding increased support was suggested for troubleshooting session content as well as managing time challenges for completing home mindfulness practice. Participants endorsed developing affect regulation skills and identified several advantages to Web-based delivery including flexibility, reduced cost, and time commitment.

**Conclusions:**

These findings support the viability of providing MBCT online and are consistent with prior qualitative accounts derived from in-person MBCT groups. While there is certainly room for innovation in the domains of program support and engagement, the high levels of participant satisfaction indicated that MMB can significantly increase access to evidence-based psychological treatments for sub-threshold symptoms of unipolar affective disorder.

## Introduction

Mindfulness-based cognitive therapy (MBCT) is an empirically supported intervention designed to teach participants emotion-regulation skills for reducing residual depressive symptoms and avoiding relapse triggers that contribute to a chronic illness course [1-3]. A recent meta-analysis indicated that MBCT conferred a 43% reduction in relative risk for relapse among participants as compared to controls [4]. Despite MBCT’s efficacy, it faces challenges to dissemination that are common to psychotherapeutic treatments, including: (1) service costs, waiting lists, and distance to access care [5], (2) scheduling and coordinating challenges inherent to delivery of an 8-session, 2-hour course with groups of 8 to 12 participants, and (3) a shortage of trained therapists who have competence in the delivery of CBT (cognitive behavioral therapy) and mindfulness meditation.

Web-based psychological interventions offer one solution to many of the aforementioned challenges [6-8]. They eliminate both travel and treatment waiting times, are cost effective with respect to clinician resources, increase treatment accessibility and flexibility, and empower participants to utilize self-help options [9,10]. The Web-based delivery of CBT for depression has been associated with large effect sizes in systematic reviews (*d*=.60) [8,11] and high user acceptability and satisfaction [12-14], particularly when combined with the use of reminders or live support [15,16]. A Web-based CBT program designed for partially remitted depressed patients showed lower relapse rates compared to controls when measured at 6 and 24 months post treatment [17,18]. Moreover, a Web-based program combining elements of Mindfulness-Based Stress Reduction and MBCT recently showed significant improvements, which were comparable to in-person group outcomes, on self-reported indices of perceived stress, anxiety, and depression [19].

The present study is a qualitative investigation of participants’ experiences with Mindful Mood Balance (MMB), the first 8-week Web-based treatment that features the core elements of the MBCT group program. Qualitative examination of other Web-based interventions has yielded valuable insights [14,20], particularly with respect to what is gained or lost in the translation from in-person formats. As a natural starting point, qualitative studies of MBCT groups provide an important perspective for examining the user experience of MMB [21,22]. Mason and Hargreaves (2001) [21], using both in-depth interviews and grounded theory methods, identified core themes among MBCT participants, including: relaxation, mindfulness skills, accepting attitude, discovery/surprise, group support and identification, knowledge of worsening mental state, and applying skills to everyday living. Allen et al (2009) [22] also conducted a qualitative study of MBCT and identified overlapping themes.

The present study’s primary objective was to learn about participants’ subjective experiences of the core intervention components, suggestions for improvement of program content, format, and delivery, and the experience of implementing mindfulness skills in their daily lives.

## Methods

### Mindful Mood Balance

MBCT integrates mindfulness meditation with the tools of CBT to teach participants skills for recognizing responses to dysphoric moods that can perpetuate and trigger more chronic mood symptoms. Although the mindfulness meditation and CBT content is delivered in an integrated manner across the 8 sessions, the first 4 sessions focus heavily on establishing a foundation of mindfulness practice and bringing awareness to daily activities, the body, and the breath. In Sessions 5 through 8, CBT principles figure more prominently as the focus becomes more squarely on depression. Home practice is assigned in each session and includes formal mindfulness practices (eg, sitting meditation, body scan meditation), informal mindfulness practices (eg, performing everyday activities mindfully), and CBT practices (writing a relapse prevention plan). MMB closely follows these concepts in the translation of MBCT to a Web-based platform.

**Figure 1:**
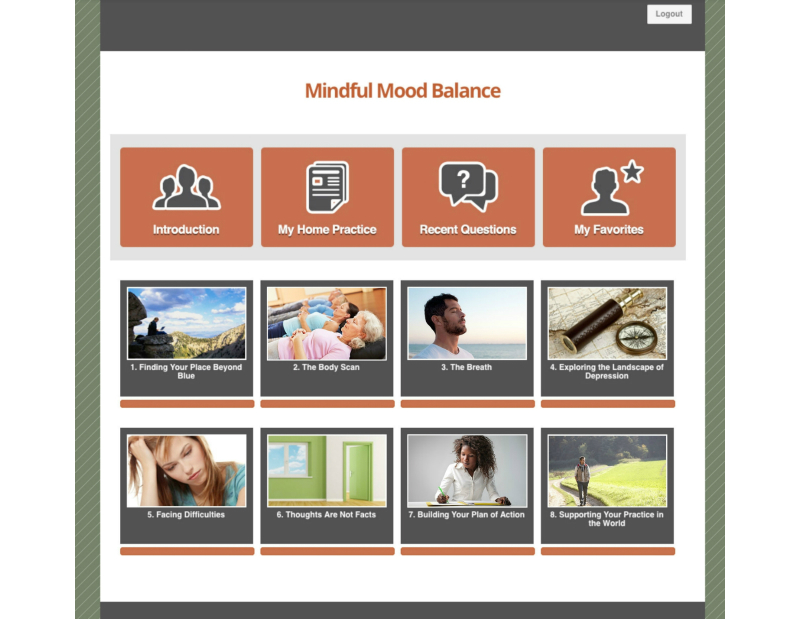
Mindful Mood Balance home page.

**Figure 2:**
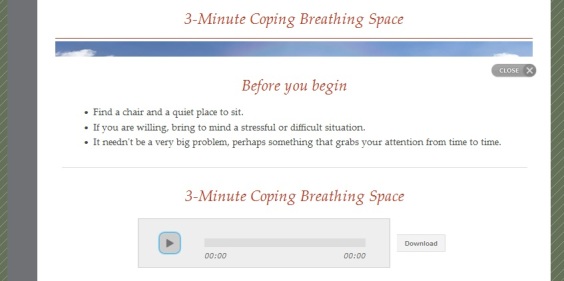
Three-minute coping breathing space completed during an MMB session with audio download available.

**Figure 3:**
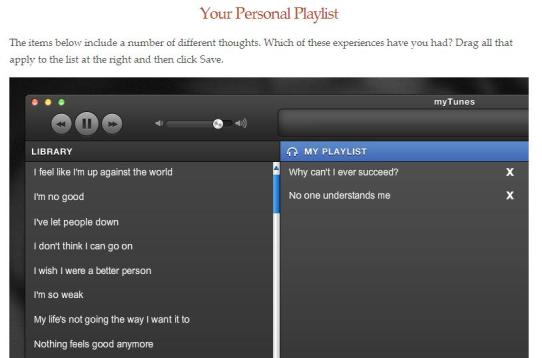
Interactive exercises - Thoughts are not facts.

**Figure 4:**
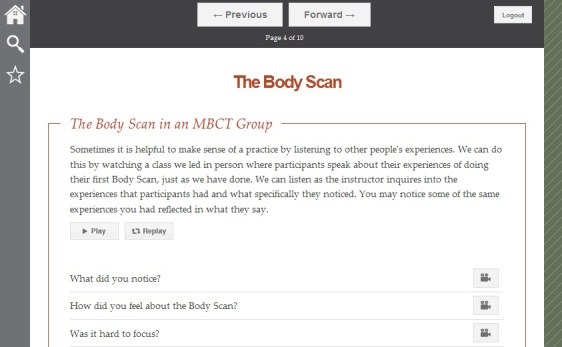
Video clips of MBCT group discussing the body scan and pertinent questions from community on this topic.

MMB used a variety of Web-based learning modalities within 60-90 minute sessions (Figure 1). First, group leaders functioned as hosts, welcoming participants to each session and guiding them through didactic content and experiential practices. Second, a substantial portion of each session was allocated to practicing mindfulness meditation (Figure 2) and engaging in a reflective process, following the practice through targeted questions that participants respond to in writing (Figure 3). Third, in order to mirror the group process component of MBCT, videos of selected portions of an in-person MBCT group were provided (Figure 4). Examples of video topics included: challenging moments during meditation, making time to actually do the practices between sessions, and reflections on implementing new ways of reacting to stressful situations. Connection with the larger group of MMB users was facilitated by an “ask a question” function, allowing all participants to anonymously post questions that were answered by MMB developers (ZVS and SD) within one week. These questions and answers were compiled across time such that participants enrolled later in the study had the advantage of viewing more content. Fourth, interactive exercises were included in the program to facilitate experiential learning of key points relevant both to mindfulness mediation practice and CBT skills. Fifth, all participants had access to a master’s level support person via phone or email (JNF), who contacted all participants within 48 hours of enrollment to introduce herself and orient them to the website and study procedures. Participants were provided with her phone number and email address and were encouraged to contact her with any questions, concerns, or challenges. Thereafter, some participants did not contact her again, while others reached out frequently via email or phone. She did not provide therapeutic intervention, but assisted with questions relating to log-in difficulties and troubleshooting both technical and situational barriers to program engagement. She also provided reminders to participants who did not log in for more than a week to help maintain engagement with the program and offer support if they were having trouble. Sixth, participants were provided with PDF and audio guides for home practice exercises, where they were asked to complete and record daily home practice using online logs. Participants were able to record several days at one time and if participants did not record home practice at least weekly, the support person provided phone or email reminders.

### Participants

All participants were Kaiser Permanente Colorado members over age 18 who provided informed consent to participate in an open trial efficacy study of MMB (N=100). This qualitative study was embedded within a larger quantitative assessment of patient outcomes for the MMB program (quantitative results to be published elsewhere). Participants were recruited from primary care and behavioral health medical settings via an invitation letter, flyer posted in medical office, or referral from provider. We enrolled 100 participants in the Mindful Mood Balance open trial and exit interviews were conducted with 38 participants, 37 of whom completed all 8 MMB sessions. Characteristics of all 100 participants are presented in Table 1, comparing those who were and were not interviewed.

**Table 1:** Demographic and clinical characteristics (N=100).

Characteristic		Interviewed (n=38) mean (SD) or n (%)	Not interviewed (n=62) mean (SD) or n (%)
Age, mean (SD)		46.89 (12.38)	47.71(10.90)
**Gender**			
	Male	11 (28.9%)	16 (25.8%)
	Female	27 (71.1%)	46 (74.2%)
**Race**			
	Asian	1 (2.6%)	1 (1.6%)
	Black/African American	1 (2.6%)	3 (4.8%)
	White	34 (89.5%)	53 (85.5%)
	Other	2 (5.3%)	5 (8.1%)
**Ethnicity**			
	Hispanic or Latino	1 (2.6%)	5 (8.1%)
	Not Hispanic or Latino	37 (97.4%)	57 (91.9%)
**Marital status**			
	Single	4 (10.5%)	11 (17.7%)
	Married or living with significant other	28 (73.7%)	41 (66.2%)
	Separated/Divorced	6 (15.8%)	10 (16.1%)
**Employment status**			
	Unemployed, homemaker, or retired	6 (15.8%)	17 (27.4%)
	Full-time	22 (57.9%)	36 (58.1%)
	Part-time	8 (21.1%)	6 (9.7%)
	Student	2 (5.3%)	3 (4.8%)
**No. of prior depressive episodes**			
	One	1 (2.6%)	11 (17.7%)
	Two	11 (28.9%)	9 (14.5%)
	Three or more	26 (68.4%)	42 (67.7%)
Antidepressant use in last year		31 (82%)	54 (87.1%)
At least 1 psychotherapy visit in last year		14 (37%)	23 (37.1%)
Past psychiatric hospitalization (past 5 years)		4 (10.5%)	5 (8.1%)

### Enrollment Criteria and Procedures

Inclusion criteria were: (1) English speaking, (2) access to the Internet, and (3) lifetime history of at least one major depressive episode, confirmed via Structured Clinical Diagnostic Interview (SCID) [23], and score of ≤12 on the PHQ-9 (Patient Health Questionnaire) [24]. The study was reviewed and approved by the Kaiser Permanente Institutional Review Boards (IRB) directly and by institutional agreement with the University of Toronto and the University of Colorado Boulder.

### Qualitative Study Design

All participants in the MMB study were invited to participate in an exit interview upon completion of the 8-session MMB open trial or upon early termination. A master’s level support person (JNF) conducted all interviews via telephone. Interviews averaged 35 minutes (ranging from 30-60 minutes). Consistent with grounded theory, an iterative process was used where the interviewer made notes after each interview, consulted periodically with the study team, and clarified key concepts in subsequent interviews.

Interview questions focused on the following areas: components of the MMB website (animations, group videos, leader videos, text, reflection questions, etc); ways MMB facilitated or hindered their ability to complete home practice; organization, clarity, quality, and quantity of information provided; and skills learned, personal insights, or information from their experience in MMB (full interview guide is available in Multimedia Appendix 1).

### Data Analysis

Interviews were de-identified, transcribed, and loaded into Atlas.ti (qualitative software analysis program) to assist with content analysis using both inductive and deductive approaches. The inductive approach applied several key concepts consistent with grounded theory including: first cycle coding, memo writing throughout data collection and analysis, categorization of codes into larger themes in order to develop a theory of the data presented, and explicitly defined saturation [25]. The first author (JMB) conducted content analysis with code review performed by CAM. Initially, all interviews were read and document summary memos were created for each transcript [26]. Thematic memos were created as themes emerged throughout summarizing and subsequent coding. The document and thematic memos were used to create a data driven code book. Next, a first cycle coding process was conducted using this code book. Throughout coding, new codes were added and clarification of code definitions was periodically performed with the members of the research team (JNF, ZVS, SD, AB) using peer debriefing sessions. These procedures yielded 54 codes with an average of 19.8 quotations per code (ranging from 5-61 quotations). Ten codes had less than 5 associated quotations and these were considered to not meet adequate saturation, due to their limited presence and lack of applicability to larger concepts under study here. A reliability check was conducted by CAM, who reviewed a sample of 5 quotations for each of the remaining 54 codes in order to evaluate the fit of each quotation to the code definition. Discrepancies of fit were discussed by CAM and JMB, some quotations were re-categorized into different codes or eliminated from the code book, and a few code definitions were re-worded to more accurately capture the quotations. When all coding was complete, the 54 process codes were organized into large categories [27]. The code book was created after extensive memo writing of the interview data, which resulted in thorough code definitions. This catalyzed the development of large categories and theories without the need for second cycle coding. Network modeling in Atlas.ti was used to create the large categories, which assists with building visual representations of code schemes. Categories included: participant feedback on MBCT Web-based content, participant feedback on MBCT Web-based group process, and participant feedback on home practice.

A separate analysis was undertaken for a code with the largest number of quotations (186) called “evidence of concept comprehension”. This analysis used evaluation coding or a priori code definitions from Allen et al (2009) who reported participant experiences with in-person MBCT using thematic analysis. The purpose of this evaluation coding was to assess the presence or absence of learned behaviors and strategies previously described for in-person MBCT groups.

## Results

### Evidence of Concept Comprehension

The “evidence of concept comprehension” theme captured participants’ descriptions of the key concepts of MBCT and is based on 186 quotations coded in response to questions about skills and insights learned from the program.

Nearly every participant mentioned the impact of newly learned activities and tools on managing distressing thoughts and feelings associated with symptoms of depression. Many participants reported positive impacts of completing some form of daily mindfulness practice including: increased awareness of rushing through life on “autopilot” and recognition of the benefit of “slowing down”, reducing stress and anxious feelings, re-framing negative thoughts about activities they do not enjoy such as “doing diapers”, and increased awareness of emotions and their impact on behavior as shown here, “I think looking at patterns of thinking, black and white thinking and catastrophizing—to catch myself on those, rather than just reacting.” One participant described the experience of doing the home practice as follows:

A little tricky at first because it’s hard to slow things down. I was very impatient in the beginning, like, when is this going to be over? I’m not sure why, but the further I got into it, the more I started to realize it is not about rushing through, it’s more about helping me not be on autopilot anymore.

Many participants found that applying the combination of mindfulness meditation and CBT skills increased their sense of control and confidence in preventing the return of future depressive symptoms. Some participants expressed an intention to use the tools regularly as a prevention strategy, even in the absence of any signs of depression, and others indicated they would apply these tools when they felt the “creeping signs of depression”. Related to this was “knowing my triggers”, which was characterized by increased awareness and recognition of thoughts, emotions, or behaviors that signal relapse. Participants reported that mindfulness meditation practices taught them to “stay focused on the present moment”, and as described by one participant, helped to recognize early warning signs: “I always think I’m over it and then it sneaks up on me. I think this—staying focused on the present moment—would help me recognize sooner what was going on.”

The CBT portion of the program and its emphasis on teaching “thoughts are not facts” was often mentioned in regard to observing, questioning, and objectifying depressive thoughts and emotions (ie, viewing these thoughts and emotions as characteristics of depression, not as representative of the self or “truth”), thereby reducing their impact. One participant expressed the concept of “thoughts are not facts” this way:

One of my triggers or clues that I’m going to possibly start having problems is having images of horrible things happening—that’s always been one of my big things—thoughts that come out of nowhere, and so because of that I’m able to say, ‘That’s not real. That’s a thought.’ And that’s been really helpful, rather than, ‘Why am I thinking that!’ So it’s sort of just accepting it and moving on.

Some participants reported changing their focus from negative to positive during the MMB program by engaging in such positive activities as exercise, enjoying the outdoors, socializing, being more self-compassionate, or focusing daily on a positive experience. The positive impact of the MMB experience is described below:

I think it gives me a sort of foundation of experience and after committing to it and doing my best with it, I can remember back—I was a happier more grounded person when I was doing that (MMB). So I always know there’s a tool that I can use to make life a little more enjoyable.

### Participant Feedback on MBCT Web-Based Content

Almost all participants thought that the text was clear and concise and that it conveyed key concepts effectively. Approximately half the participants chose to have the text on webpages read aloud and even those who did not use this audio feature believed that having this option was important. The audio to text option created a different way of interacting with the Web-based program that is described well by this participant:

I thought that [audio to text] was very helpful. By the end, I was choosing to do that instead of reading. I found that listening probably put the emphasis on the words in places it was meant to be rather than my interpretation of the words. Perhaps they didn’t always match. I’m a really literal person, so sometimes I lose things. It was a deeper understanding for me to listen to it. And if there was something I missed or didn’t think I understood, I could go back and read it, but I think having both is useful.

The videos of the group leaders speaking as hosts of the program for participants were received very positively and some participants believed this was a good alternative to written text for conveying information, although some preferred to read text. The videos included motivational content about accepting and being gentle with oneself in the learning process and also addressed common challenges associated with learning to practice mindfulness meditation. Specific topics included self-criticism, impatience, or feelings of inadequacy and guilt. Some participants expressed neutral responses to the group leader videos, commenting that they were “fine” or “good” or “just transitional”. Others found more value in the videos and reported that the group leaders were highly qualified and trustworthy. “I could tell they were both very empathic and very invested and believed in it. They felt very authentic and felt like they were really practitioners. I liked both of them a lot.”

Participants strongly supported the PDF worksheet downloads for home practice and the MP3 audio downloads of meditation practices. Several commented that this provided them with specific tools to retain after the program ended that could support ongoing practice. “I think having downloaded some of the PDFs will be a good resource too, so if I feel I might be heading toward a relapse, I will have something active to do.” Others felt the printed PDF increased accountability by providing a daily reminder. Some downloaded the MP3 files to their smartphone, so they could access the meditation practices at any time. There were suggestions for improvements to the PDFs and downloads including availability on iTunes, smartphones, integrated calendar reminders, adding visuals to the worksheets, and a “take-home kit” or list of all PDFs and MP3s in one place at the end of the program (in addition to weekly sessions).

Interactive exercises and animations were included to convey key concepts and metaphors, such as watching thoughts as if they were “leaves” floating down a river. Interactive exercises included developing a “playlist” of negative automatic thoughts that individuals frequently struggled with when depressed. Most people reported that they did not remember the animations during the exit interviews or find these to be especially powerful as a learning device. When reminded of examples, some still had trouble recalling them and comments were mostly neutral (eg, “they were fine”) or negative (eg, “superfluous”).

### Participant Feedback on Web-Based MBCT Group Process

Converting an in-person group program to a Web-based format, where the experience is inherently individual, presents an interesting challenge. Benefits of the Web-based format were noted as: the flexibility of completing weekly sessions on one’s own schedule, the ability to temporarily pause the sequence of sessions for travel and other reasons, freedom from pressure to drive to classes or find child care, and feeling less self-conscious doing the program from home.

Participants identified live interactivity as a key difference between these two formats. For a number of participants who had previously participated in a mindfulness group, the Web-based approach lacked some advantages of the in-person format. Chief among these were the absence of an instructor and the opportunity to learn together as a group. “It really wasn’t as satisfying as an in-person group, like questions on this sort of thing aren’t normally linear, but you fumble along and the leader answers and there is back and forth.”

The embedded videos of an in-person group, a central feature of MMB, were well received and participants indicated that they felt a connection to the group members. Participants appreciated that the same group was featured in every session, so that they got to know the individuals and develop a sense of belonging. A key advantage identified from the group videos was the knowledge that they were not the only ones who found the material and mindfulness practices challenging. “What I liked about them (the group videos) is that I went through the exercises and if I wasn’t sure what it meant or what I felt about it, I watched the group and it made more sense.” Others indicated that the group members’ adaptations of home practice to fit with the demands of their lives provided important insights. Not all reactions to the group videos, however, were positive. Some participants thought that there were too many videos, that it took too long to watch all of them, and that they should watch every single clip. However, others were more flexible and were selective in watching only those videos that were relevant to their experience. Additionally, a small subset of participants indicated they were not “group learners” and did not find the group videos to be an important aspect of their learning. One participant commented, “If I ever needed counseling or therapy of some sort, the last thing I would do is go to a group.” Another expressed, “I really didn’t watch a lot of them…I tend to look at my depression as more of an individual situation. I just could not relate to them and what they were doing and what they were feeling.”

When asked about ways to improve MMB, many participants described the desire for actual interaction with other MMB participants or a therapist. “I think this would be a lot better if there was a Web-based group…I felt alone out here. I would have been engaged more.” Some described needing more support as they went through the program, “I needed my hand held a little more.” Others described a desire for feedback from a professional therapist in person and some suggested professional feedback could be provided to reflection question inputs. Some saw the “Ask a Question” function as a good way to have some interaction beyond the website and enjoyed posting or just reading materials in this section posted by other users. Suggestions were provided for adding a community component such as: Web-based message boards with other users, hold a few in person meetings (although the logistical scheduling barriers to this approach were recognized), or create opportunities for telephone and/or video chat meetings.

Interestingly, all participants saw the value of having a support person available who was only a phone call or email away. Some participants mentioned more frequent interactions with the support person and even those who did not use the support reported that it was an important asset of the program. The benefit of reminders and follow-up contacts also was mentioned: “I think it’s nice to have someone available, whether someone wants it or not. It was nice that you were like, ‘you haven’t logged on in a while’…it’s hard to stay on track.”

### Participant Feedback on Home Practice

Participants described distinct preferences regarding the home practice component of MMB. While there was broad endorsement of the 3-minute Breathing Space, reactions to the Body Scan was more mixed. Positive responses noted that it was an effective relaxation technique, particularly for helping to “wind down” at the end of the day, whereas others reported that they felt uncomfortable with it.

Overall, there was a very well-saturated theme of time and commitment challenges for home practice. Most preferred the shorter practices for several reasons, including: scheduling time, attention or focus drifting with longer practices, impatience or trouble slowing down the mind or sitting still, or difficulty finding time alone for practice. One suggestion to address home practice challenges was a slower “ramp-up” to the longer practices. For example, it was suggested that the program should start with a 15-minute meditation, not a 30-minute meditation. However, some participants reported that they preferred the longer practices because they allowed time for the mind to slow down and focus. Additionally, some believed that the expectation for time commitment was unreasonable and felt guilty or resentful that they could not meet it. For example, in response to a question about her experience with home practice, this participant describes the self-criticism she experienced as well as some benefit from the practice: “[I felt] a little critical of self, felt like I couldn’t do it all, and it was my fault somehow, and this is too much to ask with your daily life, and resentful. But I tried my best to do it all, gave it a pretty good effort, and got stuff out of it.”

Others saw the value in completing the home practice and understood that the time investment is important to learn a new skill. The balance of positive and negative feedback was articulated by one participant:

It was difficult in that you had to carve out the time really consistently, but it was also really valuable. I don’t think the program would be as effective if you weren’t being asked to do it daily. What I understand is you’re trying to develop a habit. My only suggestion would be to give the option to do 15-20 minute practices and stress that any time they can do the 30 minutes, they should.

## Discussion

### Evidence of Concept Comprehension: Comparison to In-Person Delivery

We found a high degree of endorsement in the evaluation coding for themes previously identified by others describing learned behaviors and strategies associated with in-person delivery of MBCT [21,22]. Specifically, we found participants had increased awareness for personal warning signs of impending relapse and that they utilized meditation practices and/or cognitive behavioral strategies like “thoughts are not facts” to counteract these symptoms. We also observed that some participants used the meditation practices regularly to assist them daily with residual depressive symptoms. Furthermore, we observed that participants felt an increased sense of personal agency to handle depression and also reported increased engagement in positive activities. Contrary to prior work, we found little evidence of themes indicating improved relationships, increased self-value, or decreased feelings of isolation. One explanation for this might be the absence of synchronous group interaction in MMB compared to in-person delivery.

While MMB attempts to provide an experiential learning experience through videos of an in-person group presented in each session, it is to be expected that there will be some loss of the interpersonal learning dynamic in a Web-based program. It is possible that enhancing Web-based opportunities to engage with other MMB learners may improve the saturation of interpersonal learning in this program. The addition of a community of participants learning MMB, either synchronously or asynchronously, may help foster skills relevant to valuing self, feeling closeness with others, and it may also help participants feel less isolated.

### Enhancing the Support Function and Re-Purposing Content for Delivery Online

A noted limitation of the Web-based program was the loss of direct contact with instructors compared to in-person groups and the reduced accountability for completing sessions and home practice. These are important issues to address for any Web-based program [28], but especially so for MMB, as the likelihood of clinical benefits are linked to the frequency of practice [29]. Although we were unable to collect non-completer exit interviews, we know that the most frequently cited reasons for dropout were time/scheduling issues. It will be important to enhance and clarify MMB’s messaging at the outset, regarding time expectations for session and home practice completion in order to improve participant satisfaction and increase retention. Participants offered a number of constructive suggestions for addressing this concern: a slower ramp-up each week of the time commitment for home practice exercises, options for short or long home practice exercises each week, addition of a community function with other users or a therapist to maintain engagement, keeping the live support person, better integration of program content with personal technologies (calendar reminders, iTunes accessibility, ability to view MMB on iPad or smartphone), and outlining expectations for degree of session participation (eg, you don’t have to watch every video).

Considering that participants perceived a lack of support, it is not surprising that there were suggestions to curtail the frequency or duration of home practice—something reported in traditional MBCT groups as well. Prior studies of Web-based interventions suggest that reminders and administrative or therapist support are associated with higher retention rates and satisfaction with Web-based depression treatments as compared to unsupported delivery formats [15,16,30]. There was strong endorsement for the live administrative support and reminders in MMB. While participants were informed of the home practice time commitment during the consent process, perhaps there is a misconception that an online program implies a lesser time commitment. It will be important for future iterations of MMB to refine the support function, potentially by adding an online community, and also to clarify the importance and expectations for home practice at the outset. Other Web-based depression treatment programs have offered therapist support, although the use of professional therapists may be cost prohibitive when planning for more widespread dissemination of these programs [13,14].

### Balancing Flexibility and Fidelity

Our results indicated differing and sometimes contradictory preferences, such as the desire by some for increased group interaction versus a lack of interest in the group dynamic. Other differences were a matter of preference, such as reading text to understand the content versus listening to audio or watching a video. These differences highlight the importance of offering flexibility in order to appeal to a large audience of users, while still holding true to the MBCT model. The experiences of the “non-group learners” merit consideration as they demonstrate that completing MBCT online could be different, but still potentially beneficial. Other issues of fidelity include balancing participants’ desire to reduce the amount of home meditation practice, in light of the empirical support for a link between degree of home practice and improved clinical outcomes [29]. These are but two of a number of issues that are likely to arise when adapting the online version of MBCT to a wider and more diverse population.

### Integration With Personal and Mobile Technologies

The PDF downloads of home practice exercises and MP3 downloads of meditation practices were among the most popular features of MMB. Enhancements of these features were suggested including a compilation of downloads so participants would have a tangible take-home tool kit as a reference in case they sensed depression returning in the future. Additionally, participants wanted better integration with their iTunes accounts for MP3 meditation practice downloads, integrated calendar reminders, and also suggested access to the entire program from smartphones or iPads.

The strong desire to have an organized home tool kit and more seamless integration with current personal use technologies illustrates participants’ preferences for continued support structures that would help to sustain recovery. Specifically, participants desired better integration of program content with daily habits to promote mindfulness practice after program completion. This feedback is valuable for future website modifications to MMB and may also be pertinent to others developing online psychological treatments.

### Limitations

This qualitative investigation has several limitations that should be considered. Although we sought feedback from both completers and non-completers, we were successful only in gaining one interview from a non-completer. Therefore, the feedback presented represents a population of engaged participants; non-completers may have expressed more negative reactions to MMB, which are not represented here. Additionally, interviews were collected shortly after program completion and, therefore, we do not know if the concepts discussed would reflect participants’ appraisals of MMB and their use of learned skills over a longer time period. Our sample was relatively homogenous with regard to race and ethnicity and it included a number of individuals with prior experiences and interest in mindfulness practices. This may limit the generalizability of the viewpoints expressed to more diverse populations. The potential for subjective bias was possible with one primary coder (JMB) who was closely involved in project management and recruitment for the MMB trial; however, the coding review by CAM provided alternative perspectives that were included in the final code book. It is also possible that social desirability biased some responses given that the research assistant (JF) who conducted the interviews was associated with the MMB program through her role as the administrative support member; however, it is possible that her relationship with participants and familiarity with the MMB program facilitated more detailed disclosure. Since this is the first evaluation of online provision of MBCT, our findings should be considered tentative until qualitative analyses of new cohorts are available.

### Conclusions

The current study describes participant experiences with MMB, including challenges, positive elements, and suggestions for programmatic changes to increase engagement and effectiveness. Some of the feedback presented here may inform future studies with similar types of Web-based depression intervention programs, particularly the need to define participation expectations, include options for different levels of time commitment and to respond to desires for increased group support and integration with personal use devices. The close alignment between participants’ reports of skills learned in MMB and those that are central to the in-person MBCT program also may provide encouragement for the translation of other in-person psychological treatments to a Web-based platform. As identified here, balancing fidelity to the traditional MBCT model with participants’ desire for flexible modes of learning will need to be carefully negotiated. We foresee this challenge as vital for any future studies in this area. As a brief Web-based program, MMB may offer a scalable, cost-effective alternative to in-person MBCT that may be effective in reducing residual depressive symptoms and preventing depressive relapse.

## Multimedia Appendix 1

Interview guide.

## Abbreviations

CBT: cognitive behavioral therapy
MBCT: mindfulness-based cognitive therapy
MMB: Mindful Mood Balance
PHQ-9: Patient Health Questionnaire
