# Supplementary material for: Web-Based Intervention in Mindfulness Meditation for Reducing Residual Depressive Symptoms and Relapse Prophylaxis: A Qualitative Study
Source: J Med Internet Res. 2014 Mar 24;16(3):e87. doi: 10.2196/jmir.3129 (PMC3978551; doi:10.2196/jmir.3129)
Supplement: Supplementary file 1 [file jmir_v16i3e87_app1.pdf]

Appendix 1 - Interview Guide

1. Please tell us about the issues you experienced with the following features, and ways in which the following features could be improved upon: videos of group, text, animations, videos of group leaders, mindfulness practices, audio, PDF worksheets, reflection questions.
2. What material did you have trouble understanding? What was difficult to understand about it? In what ways could this material have been easier to understand?
3. What issues did you have with how the material was organized? In what ways could the material have been better organized?
4. What difficulty did you have using this site? In what ways could this site have been easier to use?
5. Did you make any "mistakes" on the website? In what ways, if any, was it difficult to recover from these mistakes?
6. What kind of information did you feel you needed that wasn't provided on the website? Did you look for that information on the website? Where?
7. What did you expect you would learn from this website? How was the information provided different from what you expected?
8. How will the site be useful for you in staying well over time? How will it not be useful?
9. How likely are you to use the information you learned on this website? In what ways? For what reasons would you not be likely to use the information and skills provided?
10. Please describe the biggest problems you had with this course. Why was this problematic?
